# Serine/arginine-rich splicing factor 3 (SRSF3) regulates homologous recombination-mediated DNA repair

**DOI:** 10.1186/s12943-015-0422-1

**Published:** 2015-08-19

**Authors:** Xiaolong He, Pei Zhang

**Affiliations:** Department of Biopharmaceutical Sciences, College of Pharmacy, University of Illinois at Chicago-Rockford Campus, 1601 Parkview Avenue, Room N308, Rockford, IL 61107 USA; University of Illinois Cancer Center, University of Illinois at Chicago, Chicago, IL USA

## Abstract

**Background:**

Our previous work found that serine/arginine-rich splicing factor 3 (SRSF3) was overexpressed in human ovarian cancer and the overexpression of SRSF3 was required for ovarian cancer cell growth and survival. The mechanism underlying the role of SRSF3 in ovarian cancer remains to be addressed.

**Methods:**

We conducted microarray analysis to profile the gene expression and splicing in SRSF3-knockdown cells and employed quantitative PCR and western blotting to validate the profiling results. We used chromatin immunoprecipitation to study transcription and the direct repeat green fluorescent protein reporter assay to study homologous recombination-mediated DNA repair (HRR).

**Results:**

We identified 687 genes with altered expression and 807 genes with altered splicing in SRSF3-knockdown cells. Among expression-altered genes, those involved in HRR, including BRCA1, BRIP1 and RAD51, were enriched and were all downregulated. We demonstrated that the downregulation of BRCA1, BRIP1 and RAD51 expression was caused by decreased transcription and not due to increased nonsense-mediated mRNA decay. Further, we found that SRSF3 knockdown impaired HRR activity in the cell and increased the level of γ-H2AX, a biomarker for double-strand DNA breaks. Finally, we observed that SRSF3 knockdown changed splicing pattern of KMT2C, a H3K4-specific histone methyltransferase, and reduced the levels of mono- and trimethylated H3K4.

**Conclusion:**

These results suggest that SRSF3 is a new regulator of HRR process, which possibly regulates the expression of HRR-related genes indirectly through an epigenetic pathway. This new function of SRSF3 not only explains why overexpression of SRSF3 is required for ovarian cancer cell growth and survival but also offers a new insight into the mechanism of the neoplastic transformation.

**Electronic supplementary material:**

The online version of this article (doi:10.1186/s12943-015-0422-1) contains supplementary material, which is available to authorized users.

## Background

Serine/arginine-rich splicing factor 3 (SRSF3), previously named as SRp20 and SFRS3, is the smallest member of serine/arginine-rich (SR) protein family, well known for its regulatory roles in RNA metabolism and functions, such as pre-mRNA splicing [1–4], mRNA 3′ end processing [5, 6], mRNA export from nucleus [7–9] and cap-independent translation [10, 11]. SRSF3 was also implicated in the regulation of chromatin structure and function because of its association with interphase chromatin but not with hyperphosphorylated mitotic chromosomes [12].

Physiologically, SRSF3 is essential for embryo development since *Srsf3*-null mouse embryos failed to form blastocysts and died at the morula stage [13]. Mice with hepatocyte-specific knockout of *Srsf3* exhibited altered hepatic architecture, prolonged expression of fetal liver markers, impaired glucose homeostasis and reduced cholesterol synthesis, suggesting that *Srsf3* is indispensable for hepatocyte maturation and metabolic function in mice [14].

Pathologically, there is increasing evidence indicating that SRSF3 plays an important role in tumorigenesis. In a mouse model of mammary tumorigenesis, it was observed that SRSF3 was remarkably increased during the development of mammary cancer [15]. In human ovarian tumors, we found that SRSF3 was overexpressed in invasive ovarian cancer at all stages and its overexpression was critical for tumor cell growth and maintenance of transformation properties [16, 17]. Knockdown of SRSF3 expression causes growth inhibition or apoptosis of ovarian cancer cells, depending on the extent of SRSF3 knockdown [16]. SRSF3 was also found upregulated in a variety of other tumors, such as cervical cancer and rhabdomyosarcoma [18]. It was showed that ectopically expressed SRSF3 promoted cell growth and transformation of human and mouse fibroblasts [18]. In addition, knockdown of SRSF3 resulted in G1 arrest and downregulation of several G1/S transition-related genes in colon cancer cells [19] and led to p53-dependent cellular senescence in fibroblasts [20]. Besides the tumor promoting role, a recent study found that SRSF3 might function as a suppressor of hepatic carcinogenesis, because mice with hepatocyte-specific knockout of *Srsf3* invariably developed hepatocellular carcinoma at late ages [21].

Our previous studies mentioned above raise questions why SRSF3 is required for ovarian cancer cell growth and how it contributes to the neoplastic transformation. In the present study, we show that knockdown of SRSF3 suppresses expression of breast cancer 1, early onset (BRCA1), BRCA1 interacting protein C-terminal helicase 1 (BRIP1), and RAD51 recombinase (RAD51). These genes all play important roles in the homologous recombination (HR)-mediated DNA damage repair pathway [22, 23]. Correspondingly, we observed impaired HR-mediated DNA damage repair (HRR) activity and accumulation of DNA double-strand breaks (DSBs) after SRSF3 knockdown. We also provide evidence suggesting that SRSF3 possibly regulates the expression of above genes through an epigenetic pathway.

## Results

### Profiling of gene expression and splicing in SRSF3-knockdown cells

**Fig. 1:**
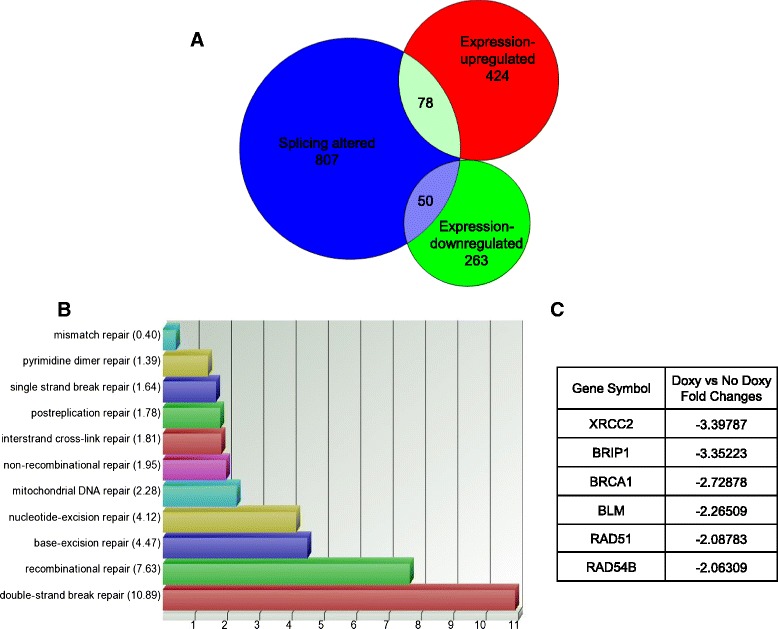
Knockdown of SRSF3 alters expression and splicing of hundreds of genes. **a** Venn diagram of expression-altered genes and splicing-altered genes. **b** Bar chart of enrichment scores under the term of DNA repair generated by gene ontology analysis. **c** List of expression-altered HRR-related genes

**Fig. 2:**
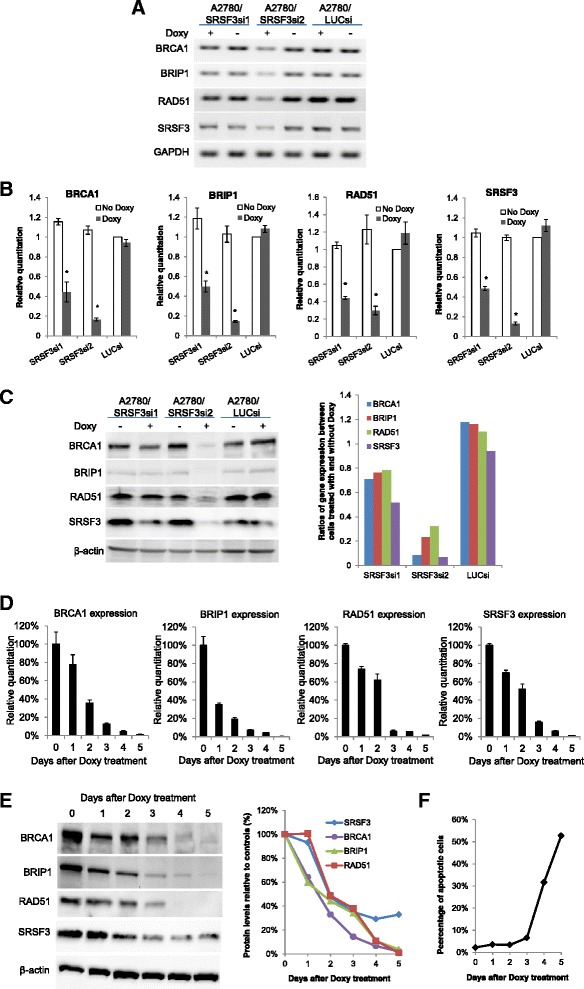
Knockdown of SRSF3 suppresses the expression of BRCA1, BRIP1 and RAD51. **a** Regular PCR amplification of cDNA fragments of BRCA1, BRIP1, RAD51, SRSF3 and GAPDH. **b** Relative quantitation of BRCA1, BRIP1, RAD51 and SRSF3 expression determined by qPCR analysis. Shown are the results of three independent experiments (mean ± s.d.). * indicates *p* < 0.01 for comparisons between samples treated with and without Doxy. **c** Left: Western blotting results of whole cell lysates of A2780 subline cells treated with or without Doxy. Right: Quantitation of the western blotting results. Results in (**a**), (**b**) and (**c**) were obtained from cells treated with or without Doxy for 3 days. **d** Time course of BRCA1, BRIP1, RAD51 and SRSF3 expression at mRNA levels determined by qPCR. Day 0 represents cells that were not treated with Doxy. Two independent experiments were performed and produced similar results. Shown are the results of one experiment (mean ± s.d. of triplicate PCR reactions). **e** Time course of BRCA1, BRIP1, RAD51 and SRSF3 expression at protein levels. Left: Western blotting results; Right: Quantitation. **f** Time course of apoptotic cells

In our previous study, we established three A2780 sublines, A2780/SRSF3si1, A2780/SRSF3si2 and A2780/LUCsi, which express doxycycline (Doxy)-induced SRSF3 siRNA1 (SRSF3si1), SRSF3 siRNA2 (SRSF3si2) and luciferase siRNA (LUCsi), respectively. SRSF3si1 and SRSF3si2 suppress SRSF3 expression by about 50 and 90 %, respectively, while LUCsi has little effect on SRSF3 expression [16]. We confirmed these results in the present study by regular reverse transcription PCR (RT-PCR), quantitative RT-PCR (qPCR) as well as western blotting, as shown in the Fig. 2a, b and c. Induction of SRSF3si1 caused cell growth inhibition whereas induction of SRSF3si2 led to apoptosis [16] (Fig. 2f). In order to determine the mechanisms underlying the role of SRSF3 in ovarian cancer, we conducted human exon microarray analysis to examine the genome-wide profiles of gene expression and splicing in A2780/SRSF3si2 cells with or without SRSF3 knockdown. Using *p* < 0.05 and absolute fold changes greater than 2 as the cutoff values, we found 687 genes altered in their expression in SRSF3-knockdown cells, among which 424 genes were upregulated while 263 genes were downregulated (Additional file 1: Table S1). Using false discovery rate (FDR) less than 0.05 as the criterion, we identified 807 genes altered in their splicing in the SRSF3si2 cells (Additional file 2: Table S2). Shown in Fig. 1a is the Venn diagram of expression-altered genes and splicing-altered genes in SRSF3-knockdown cells. Gene ontology analysis revealed that genes involved in double-strand break repair, especially those involved in HRR, were enriched among the expression-altered genes, as shown in Fig. 1b. Figure 1c lists the changed HRR-related genes, which are all downregulated in SRSF3-knockdown cells. In addition, genes involved in sterol biosynthesis are also enriched in the expression-altered genes and they are all upregulated in SRSF3-knockdown cells (Additional file 3: Figure S1). Among the splicing-altered genes, those involved in cellular protein modification, especially those related to polyubiquitilation, are the most highly enriched (Additional file 3: Figure S2).

### Knockdown of SRSF3 suppresses the expression of BRCA1, BRIP1 and RAD51

We have confirmed the downregulation of BRCA1, BRIP1 and RAD51 expression induced by SRSF3 knockdown at both mRNA and protein levels, as shown in Fig. 2. We confirmed the downregulation of other three genes, XRCC2, RAD54B and BLM, only at mRNA levels (Additional file 3: Figure S3) but not at protein levels due to problems with the antibodies we tested. Figure 2a and b show the results of RT-PCR and qPCR, respectively. Figure 2c shows the results of western blotting. As can be seen, the downregulation of BRCA1, BRIP1 and RAD51 is more substantial in Doxy-treated A2780/SRSF3si2 cells than in Doxy-treated A2780/SRSF3si1 cells, indicating that the effects correlate with the extent of SRSF3 knockdown. As the primer pairs used for PCR are located on the exons common to all or most known splice variants of these genes, the results shown in Fig. 2 reflect the downregulation of overall expression rather than specific splice variants. Similar results were obtained with sublines of another ovarian cancer cell line, SKOV3, as shown in the Additional file 3: Figure S4, indicating that the phenomenon is not cell line specific. It is worth pointing out that our microarray analysis did not find any significant alterations in the splicing of BRCA1, BRIP1 and RAD51 in SRSF3-knockdown cells.

We also measured the time course of the expression of BRCA1, BRIP1 and RAD51 at mRNA and protein levels after the A2780/SRSF3si2 cells were treated with Doxy. As can be seen in Fig. 2d and e, the expression of these genes started to decrease from day one after Doxy treatment and the downregulation was gradually intensified in the later days. These results suggest that downregulation of these genes is likely a primary effect of SRSF3 knockdown rather than secondary to the growth inhibition or apoptosis caused by SRSF3 knockdown, which was not observed until day 4 after Doxy treatment, as shown in Fig. 2f.

### SRSF3 knockdown-induced downregulation of BRCA1, BRIP1 and RAD51 is not due to nonsense-mediated mRNA decay (NMD)

**Fig. 3:**
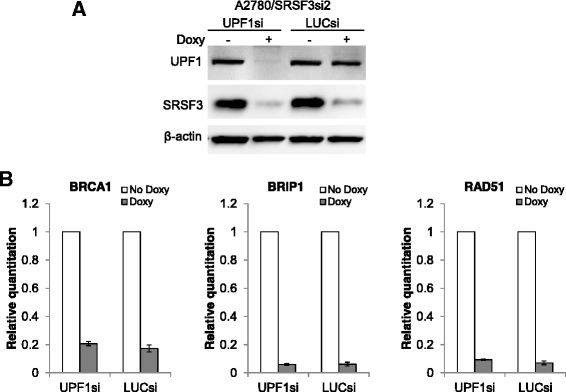
SRSF3 knockdown-induced downregulation of BRCA1, BRIP1 and RAD51 is not due to NMD. **a** Western blotting results showing Doxy-induced simultaneous knockdown of UPF1 and SRSF3. **b** Expression of BRCA1, BRIP1 and RAD51 after simultaneous knockdown of UPF1 and SRSF3. Shown are the results of three independent experiments (mean ± s.d)

NMD is an important quality-control mechanism but also plays a role in the regulation of gene expression. It recognizes and degrades mRNAs harboring premature termination codons (PTCs) [24]. SRSF3 is a well-known splicing factor and its knockdown may cause aberrant splicing and thus trigger NMD to downregulate gene expression. To determine whether the downregulation of BRCA1, BRIP1 and RAD51 is mediated by this mechanism, we examined the effects of inhibition of NMD pathway on the expression of these three genes. NMD is primarily carried out by up-frameshift (UPF) proteins, which consist of UPF1, UPF2 and UPF3 with UPF1 as the key effector of NMD [25]. Previous studies showed that depletion of UPF1 by shRNAs substantially inhibited NMD activity, leading to the upregulation of hundreds of mRNAs [26, 27]. We introduced one of the reported UPF1 siRNA (UPFsi) sequences [28] or LUCsi sequence [29] into A2780/SRSF3si2 cells using lentiviruses and achieved Doxy-induced simultaneous knockdown of SRSF3 and UPF1, as shown in Fig. 3a. Then we measured the expression of BRCA1, BRIP1 and RAD51 in these cells treated with or without Doxy for 3 days by qPCR. As can be seen in Fig. 3b, these genes were similarly downregulated in A2780 cells simultaneously expressing SRSF3si2 and UPF1si or SRSF3si2 and LUCsi, indicating that the downregulation of these genes could not be reversed by inhibition of NMD and thus was not mediated by NMD.

### Knockdown of SRSF3 suppresses the transcription of BRCA1, BRIP1 and RAD51

**Fig. 4:**
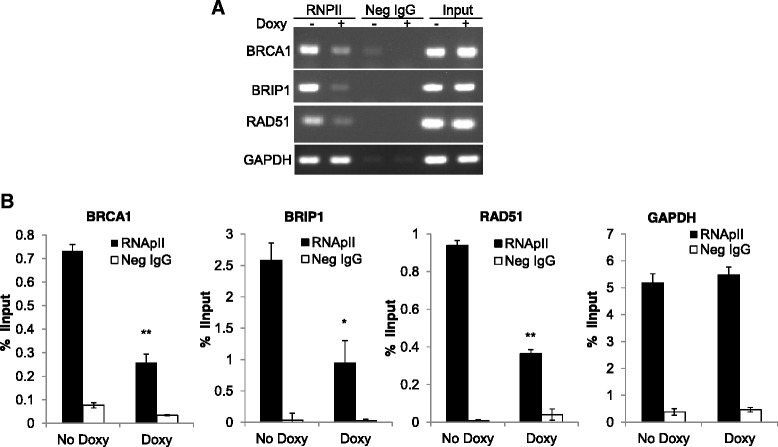
Knockdown of SRSF3 reduces RNApII occupancy on BRCA1, BRIP1 and RAD51 genes. **a** Regular PCR amplification of immunoprecipitated chromatin DNAs. **b** qPCR analysis of immunoprecipitated chromatin DNAs. Shown are immunoprecipitated DNAs expressed as percentages of corresponding input DNAs (mean ± s.d, *n* = 3). * and ** indicate *p* < 0.05 and *p* < 0.01, respectively, for comparisons of RNApII occupancy between samples treated with and without Doxy

To determine whether the downregulation of BRCA1, BRIP1 and RAD51 is caused by reduced transcription, we examined RNA polymerase II (RNApII) occupancy on these genes in A2780/SRSF3si2 cells treated with or without Doxy using chromatin immunoprecipitation (ChIP) technology. RNApII occupancy on chromatin DNA has been shown to be reliable surrogate readout for transcription rates [30, 31]. We analyzed RNApII occupancy in two regions for each gene: one is about 1 kb downstream of transcription start site (TSS) and the other is 10 kb to 14 kb downstream of TSS. Chromatin DNAs precipitated by RNApII antibody or negative control IgG (Neg IgG) were analyzed by regular PCR and qPCR. As shown in Fig. 4 (results of ChIP in the region of 1 kb downstream of TSS) and Additional file 3: Figure S5 (results of ChIP in the region of 10 to 14 kb downstream of TSS), RNApII occupancy was decreased in BRCA1, BRIP1 and RAD51 genes, but not in the control gene, GAPDH, after SRSF3 knockdown. Figure 4a shows the results of regular PCR and the Fig. 4b shows the results of qPCR.

### Knockdown of SRSF3 impairs HRR and increases DSBs

**Fig. 5:**
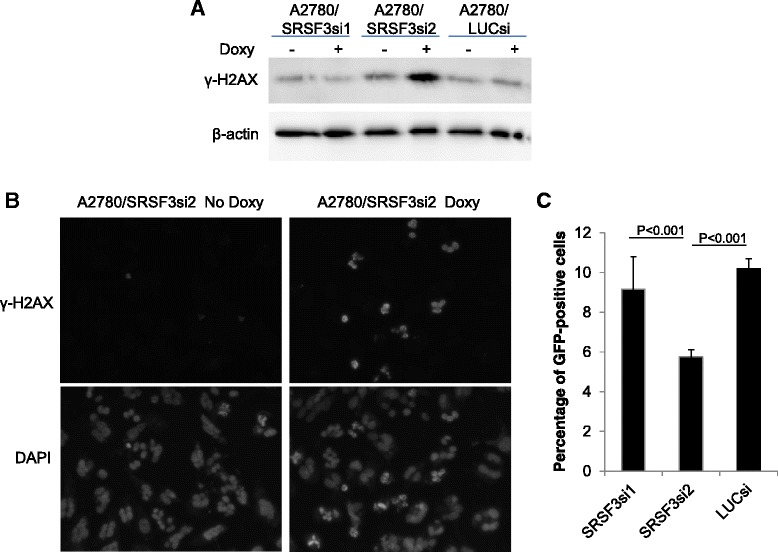
Knockdown of SRSF3 increases accumulation of DSBs and impairs HRR activity. **a** Western blotting result of γ-H2AX. Cell lysates were prepared from cell cultures treated with or without Doxy for 3 days. **b** Immunofluorescent staining of γ-H2AX in A2780/SRSF3si2 cells treated with or without Doxy for 3 days. **c** Results of HRR assays. Shown are the percentage of GFP-positive cells determined by flow cytometry (mean ± s.d., *n* = 4)

Given the role of BRCA1, BRIP1 and RAD51 in HR-mediated repair of DSBs, the downregulation of their expression is very likely to impair this process and cause accumulation of DSBs in the cells. To test this hypothesis, we first examined the levels of γ-H2AX, a biomarker of DSBs, in A2780 subline cells treated with or without Doxy by western blotting. As shown in Fig. 5a, γ-H2AX was substantially increased in A2780/SRSF3si2 cells treated with Doxy but not in other Doxy-treated subline cells, indicating that robust suppression of SRSF3, which resulted in deeper downregulation of BRCA1, BRIP1 and RAD51 (Fig. 2), indeed caused accumulation of DSBs. Immunofluorescent staining of A2780/SRSF3si2 cells treated with or without Doxy confirmed above finding, as shown in Fig. 5b. The time course of γ-H2AX levels after SRSF3 knockdown is shown in Additional file 3: Figure S6.

Next we examined whether knockdown of SRSF3 impaired cellular capability to repair DSBs via HR-mediated pathway. We employed DR-GFP reporter [32] to analyze HRR activity in the cell. The reporter consists of two tandem mutated GFP genes with one being a full-length GFP mutated to contain an I-SceI site and the other being a 5′ and 3′-truncated GFP in the downstream. A single DSB is generated in the upstream GFP gene by ectopically expressed I-Scel and can be repaired by HR with the downstream truncated GFP as the template, which results in the formation of functional GFP gene and thus GFP-positive cells. Therefore, the percentage of GFP-positive cells reflects the cellular capability to carry out HRR. We performed this assay in 293 T cells because of the high efficiency at which they can be transfected. As shown in Fig. 5c, the percentage of GFP-positive cells is lowest in 293 T cells expressing SRSF3si2, indicating impaired HRR in these cells. Although 293 T cells expressing SRSF3si1 also had lower percentage of GFP-positive cells than control cells, the difference between two was not statistically significant. These results correspond well to the changes of γ-H2AX shown in Fig. 5a and b.

### Expression of siRNA-resistant SRSF3 offsets the effects of knockdown of endogenous SRSF3

**Fig. 6:**
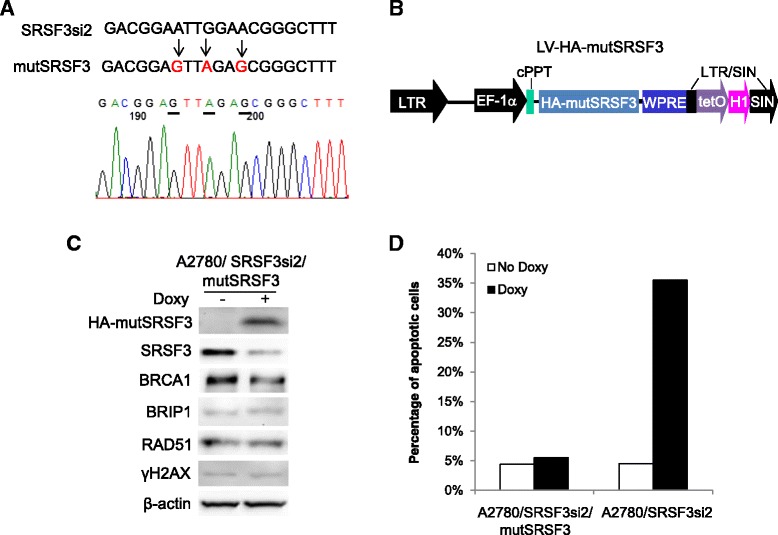
Expression of siRNA-resistant SRSF3 offsets the effects of knockdown of endogenous SRSF3. **a** Mutated coding sequence of SRSF3 that is targeted by SRSF3 siRNA2. Three silent mutations are in red. The sequencing chromatogram confirmed these mutations (indicated by the underlined nucleotides). **b** A diagram of the lentiviral vector expressing Doxy-induced HA-mutSRSF3. **c** Western blotting results. **d** Percentage of apoptotic cells

To further establish the role of SRSF3 in the regulation of HRR gene expression, we conducted rescue study to determine whether siRNA-resistant SRSF3 could offset the effects of knockdown of endogenous SRSF3 in the A2780/SRSF3si2 cells. We made three silent mutations in the coding region of SRSF3 that was targeted by SRSF3 siRNA2, as shown in Fig. 6a. The mutated entire coding sequence with HA tag fused to the N-terminus (HA-mutSRSF3) was then cloned into the lentiviral vector pLVTHM [33] under the direction of EF-1α promoter, as shown in Fig. 6b. pLVTHM was also the vector we used to express the SRSF3 siRNAs and the luciferase siRNA in the cell [16]. The expression of HA-mutSRSF3, like the expression of siRNAs, was Doxy-inducible in the cells expressing regulatory fusion protein tTR/KRAB, which is a hybrid of the tetracycline repressor (tTR) and KRAB domain of human Knox1 protein [33]. We infected A2780/SRSF3si2 cells using the lentiviruses carrying HA-mutSRSF3 expression cassette and obtained a new cell culture (A2780/SRSF3si2/mutSRSF3), which demonstrated Doxy-induced expression of HA-mutSRSF3 and simultaneous suppression of endogenous SRSF3, as shown in Fig. 6c. With these cells we observed that Doxy treatment caused little changes in the expression of HRR-related genes BRCA1, BRIP1 and RAD51, indicating that the expression of HA-mutSRSF3 offset the effects of knockdown of endogenous SRSF3 (Fig. 6c). In accordance with unchanged expression of HRR-related genes, the expression of γ-H2AX was not increased after Doxy treatment in these cells (Fig. 6c), indicating that HA-mutSRSF3 rescued DNA damages caused by knockdown of endogenous SRSF3. Further, we found that HA-mutSRSF3 also prevented SRSF3 knockdown-induced apoptosis, as shown in Fig. 6d. Taken together, these rescue experiments provide additional evidence to support a role of SRSF3 in the regulation of HRR and cell survival.

### Knockdown of SRSF3 changes splicing pattern of lysine-specific methyltransferase 2C (KMT2C, also known as MLL3) and decreases methylated histone H3 lysine 4 (H3K4)

**Fig. 7:**
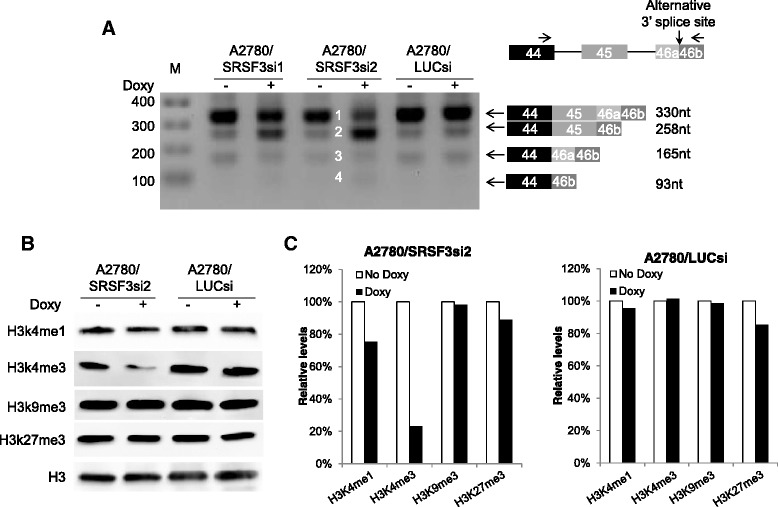
Knockdown of SRSF3 changes splicing pattern of KMT2C and decreases methylated H3K4. **a** RT-PCR of KMT2C transctipts between exon 44 and exon 46. The numbers in the middle of the image indicate the DNA bands. The diagram of the alternative splicing events is shown on the right. The right and left arrows over exons 44 and 46 represent the primer pair used for PCR. **b** Detection of monomethylated H3K4 and trimethylated H3K4, H3K9 and H3K27 by western blotting. Unmodified histone H3 was a loading control. **c** Quantitation of western blotting results in (**b**). The levels of modified H3 were first normalized to the levels of unmodified H3 and then the relative levels of modified H3 were calculated using the samples without Doxy treatment as the references

KMT2C is a H3K4-specific histone methyltransferase, catalyzing H3K4 monomethylation [34, 35]. Our exon microarray analysis found that KMT2C expression was upregulated in SRSF3-knockdown cells (Additional file 1: Table S1). According to Ensembl database, two large protein variants could be generated from this gene with one being 4911 amino acids long and the other 4968 amino acids long, depending on whether exon 45 is included. In an attempt to validate the microarray finding, we amplified the region of KMT2C cDNA spanning exon 44 to exon 46 from the samples of A2780 subline cells treated with or without Doxy. As shown in Fig. 7a, the amplification generated more DNA fragments than expected 2 DNA bands. More interestingly, SRSF3 knockdown changed the expression pattern of these fragments. Amplicon sequencing of the PCR products from A2780/SRSF3si2 cells revealed that the extra fragments were derived from the use of an alternative 3′ splice site (alt 3′ SS) within exon 46, which is located 72 nucleotides downstream of 3′ SS of exon 46. Knockdown of SRSF3 increased the use of the alt 3′ SS, resulting in substantial upregulation of splice variants (represented by bands 2 and 4 in the image of Fig. 7a) which had the 5′ portion of exon 46 (i.e. 46a in the Fig. 7a) skipped and downregulation of splice variants (represented by bands 1 and 3 in the image of Fig. 7a) containing the whole exon 46. Given the molecular function of KMT2C in H3K4 methylation, we wondered whether altered splicing of KMT2C was accompanied by any changes in H3K4 methylation. Therefore, we examined monomethylated H3K4 (H3K4me1) and trimethylated H3K4 (H3K4me3) in A2780/SRSF3si2 and the control A2780/LUCsi cells. As shown in Fig. 7b and c, H3K4me1 and H3K4me3, especially the latter, were decreased in Doxy-treated A2780/SRSF3si2 cells but not in Doxy-treated control cells. In contrast, trimethylated H3K9 and H3K27 were basically unchanged in Doxy-treated cells. H3K4me1 and H3K4me3 have been associated with active transcription [34] while H3K9me3 and H3K27me3 have been linked to gene repression [36]. Whether the downregulation of BRCA1, BRIP1 and RAD51after SRSF3 knockdown can be ascribed to the reduction of methylated H3K4 requires more investigation to determine.

**Fig. 8:**
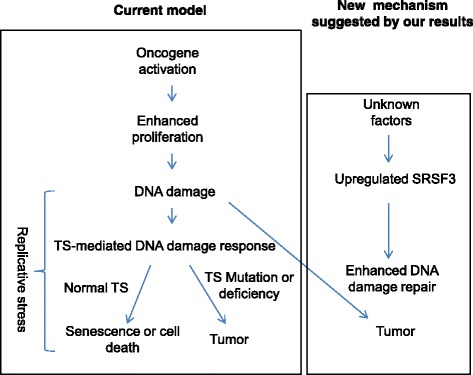
Current model and the new one suggested by the results of this study to explain neoplastic transformation. TS: Tumor suppressor

## Discussion

In this report, we present data showing that knockdown of SRSF3 results in downregulation of BRCA1, BRIP1 and RAD51 expression and causes impaired HRR activity. These results suggest a novel role for SRSF3 in the regulation of HRR pathway.

HRR is a major mechanism to repair DSBs, which are the most deleterious form of DNA damage and can be generated by exogenous insults as well as endogenous factors [37]. In dividing cells like cancer cells, DSBs are mainly caused by endogenous factors (endogenous DSBs, EDSBs), such as reactive oxygen species (ROS) and replication stress [37], and can be induced by activated oncogenes [38–41]. It was estimated that EDSBs were produced at the rate of ~50 per cell per cell cycle in the normal human cells [42]. In cancer cells, this rate could be higher because of the effects of increased oncogene activity. DSBs are repaired primarily by two mechanisms: non-homologous end-joining (NHEJ) and HRR [43, 23]. NHEJ repairs DSBs by promoting direct ligation of DNA ends, which frequently introduces insertions, deletions, substitutions and even chromosome rearrangements. In contrast, HRR repairs DSBs faithfully by using homologous sister chromatids as the template to guide the repairing process and thus playing a pivotal role in the maintenance of genomic stability [43, 23]. HRR involves following steps: DSB recognition, damage signal transduction and break repair by HR [23]. The six downregulated genes shown in Fig. 1c all have a role or roles in this repair pathway [22, 23, 44]. For example, BRCA1 helps to direct the cell to choose HRR over NHEJ to repair DSBs during S and G2 phase [44]. BRCA1 is also required for the recruitment of RAD51to the damage sites [45], which is necessary for homology search and subsequent strand exchange with intact sister chromatid duplex DNA [23].

If DSBs are left unrepaired or aberrantly repaired, the outcome would be cell death or genomic instability. Although genomic instability is a characteristic of most cancers and is believed to facilitate the development of permanent oncogenic changes in the genome [46], there is no evidence suggesting that cancer cells could tolerate continuous DNA damage generation after generation. On the contrary, a relatively stable genome is essential for any cell, normal or tumor, to grow and survive [47], and it is cancer cell’s reliance on a stable genome that makes DNA-damaging agents to be effective in cancer treatment.

Given the more frequent occurrence of spontaneous DSBs in cancer cells and the importance of a relatively stable genome for cell growth and survival, it is logical that cancer cells need upregulated HRR activity to keep their genomes from continuous alterations. Otherwise, accumulated DSBs or genomic alterations would eventually lead to cell death. The new role of SRSF3 in the regulation of HRR pathway provides a mechanism for cancer cells to meet this need. Therefore, it is no wonder that almost all invasive ovarian tumors that we examined overexpressed SRSF3 and knockdown of SRSF3 induced growth inhibition and cell death [16]. Analysis of the serous ovarian cancer microarray dataset from The Cancer Genome Atlas project shows that SRSF3, BRCA1, RAD51, XRCC2 and BLM are upregulated in tumors compared to normal ovaries, as shown in Additional file 3: Figure S7, supporting the notion that tumor cells need enhanced HRR activity.

The new role of SRSF3 discovered in this study also suggests a new paradigm to understand the tumorigenic process. It is widely accepted that activated oncogenes are a driving force of tumorigenesis [48, 49]. However, they alone cannot cause cancer. Instead, activated oncogenes induce senescence or cell death in normal and partially transformed cells due to their induction of DNA damage and DNA damage response (DDR) [49, 40]. According to current tumorigenic model, after oncogene activation, further genetic or epigenetic changes in tumor suppressor genes are needed to overcome replicative stress and make tumorigenesis proceed (Fig. 8, left panel) [48, 49]. Our observation suggests that there exist another mechanism to promote tumorigenesis. That is, during neoplastic transformation, which could be initiated by oncogene activation, SRSF3 is upregulated by presently unknown factor(s) and confers cells enhanced capability to carry out HRR and thus allows cells to bypass replicative stress and complete transformation process (Fig. 8, right panel). This new mechanism may explain not only the development of tumors that lack mutations or alterations in tumor suppressors involved in DNA damage repair and response but also the overexpression of RAD51 found in a wide variety of human tumors, including BRCA1-deficient ones [50, 51]. Overexpression of RAD51 can rescue the defects caused by depletion of BRCA1 and thus may contribute to the genesis of BRCA1-deficient tumors [51].

Finally, the results shown in Fig. 7 provide a clue to understand the molecular mechanisms behind the new role of SRSF3. Based on those results, we hypothesize that SRSF3 regulates the expression of HRR-related genes indirectly through an epigenetic pathway. That is, SRSF3 controls alternative splicing of KMT2C, whose splice variants determine the methylation status of H3K4, by which the transcriptional activities of HRR-related genes are set. To test the hypothesis, more work will be needed to establish causal relationships between the changed alternative splicing of KMT2C and reduced methylated H3K4 and between reduced H3K4me3 and suppressed expression of HRR-related genes.

## Conclusions

Our results indicate that SRSF3 is a regulator of HRR process, which possibly regulates the expression of HRR-related genes indirectly through an epigenetic pathway. This novel function explains why overexpression of SRSF3 is required for ovarian cancer cell growth and survival but also offers a new insight into the mechanism of the neoplastic transformation.

## Methods

### Cell cultures

Ovarian cancer cell line A2780 sublines, A2780/SRSF3si1, A2780/SRSF3si2 and A2780/LUCsi, were established in our previous study [16]. These sublines were grown in DMEM supplemented with 10 % FBS and 2 mM L-glutamine at 37 °C, 5 % CO_2_. 293 T cells were purchased from the American Type Culture Collection (ATCC) and grown in the same media as A2780 sublines.

### Microarray analysis

Total RNAs were extracted from A2780/SRSF3si2 cells grown in the presence or absence of Doxy (0.1μg/ml) for 3 days using TRIzol reagent (Life Technologies, Grand Island, NY) and treated with TURBO DNA-free kit (Life Technologies). The prepared total RNA samples were submitted to Asuragen (Austin, TX) for expression profiling by Affymetrix Human Exon 1.0 ST Array (Affymetrix, Santa Clara, CA). The microarray data were analyzed using Partek Genomics Suite Version 6.6 (Partek, St. Louis, MO) to determine the differentially expressed or spliced genes. Gene ontology analysis was performed also using Partek Genomics Suite Version 6.6.

### RT-PCR and qPCR

Total RNAs were extracted with TRIzol reagent from cultured cells and treated with TURBO DNA-free kit. cDNAs were synthesized from 2 μg of total RNAs with High Capacity cDNA Reverse Transcription Kit (Life Technologies). Non-quantitative RT-PCR reactions were set up with Phusion Green Hot Start II High-Fidelity DNA Polymerase (Thermo Fisher Scientific, Waltham, MA). qPCRs were set up with Fast SYBR Green Master Mix (Life Technologies) and run in StepOne Plus Real-Time PCR System (Life Technologies). The primer pairs for RT-PCR and qPCR were the same for each gene and they are BRCA1 prime pair 5′-ACTCTGAGGACAAAGCAGCG-3′ and 5′- CATCCCTGGTTCCTTGAGGG-3′, BRIP1 primer pair 5′- CGCTTTAGGAATAACCCAAGT-3′ and 5′- CTCATTGTCCTGTATATTGGTT-3′, RAD51 primer pair 5′- TTTGGCCCACAACCCATT TC-3′ and 5′- TTAGCTCCTTCTTTGGCGCA-3′, SRSF3 primer pair 5'-AATTGGAACGGGCTTTTGGC-3' and 5'-CCATCTAGCTCTCGGACTGC-3', and GAPDH primer pair 5′-GGGGCTGGCATTGCCCTCAA-3′ and 5′-GGCTGGTGGTCCAGGGGTCT-3′. The expression level of each gene was determined by the comparative CT (ΔΔCT) method [52] with GAPDH as the endogenous control and the subline A2780/LUCsi cells grown in the absence of Doxy as the reference. The primer pair for amplification of KMT2C cDNA between exon 44 and exon 46 was 5′-AGCACTGACACGTTTACCCA-3′ and 5′- AAGCCGGAGTGTTAGTGAGC-3′.

### Western blotting

Whole cell lysates were prepared with 1x sample buffer (50 mM Tris pH 6.8, 2 % SDS, 10 % glycerol, 5 % β-mecaptoethanol and 0.002 % bromphenol blue) and sonicated with Sonifier Cell Disrupters (Branson Ultrasonics, Buffalo Grove, IL). Western blotting was performed as described previously [53]. The antibodies for BRCA1, RAD51, SRSF3 and γ-H2AX were purchased from Santa Cruz Biotechnology (Dallas, TX, cat# sc-642, sc-8349, sc-13510 and sc-101696, respectively) and the antibodies for BRIP1 and UPF1 were from Cell Signaling Technology (Danvers, MA; cat# 4578S, 12040S, respectively). Quantitation of western blotting results was performed with Volume Tools program contained in Image Lab software (Bio-Rad Laboratories, Hercules, CA).

### Apoptosis assay

Cells were fixed in 4 % paraformaldehyde for 10 min and then stained in a solution of Hoechst 33342 (Life Technologies) for 15 min. Apoptotic cells and non-apoptotic cells were counted under fluorescent microscope manually with computer assistance.

### ChIP

Chromatin DNAs were isolated from A2780/SRSF3si2 cells treated with or without Doxy for 3 days and immunoprecipitated with ChIP-IT Express Enzymatic kit (Active Motif, Carlsbad, CA) and RNA polymerase II antibody (mAb) (Active Motif, Cat # 39097) or Negative control mouse IgG (Santa Cruz Biotechnology, cat# sc-2762) by following the manufacturer’s instruction. The primer pairs for non-quantitative PCR and qPCR were the same for each gene and they are following: BRCA1 primer pair, 5′-GGACGTTGTCATTAGTTCTTTGGT-3′ and 5′-TCTTCAACGCGAAGAGCAGA-3′; BRIP1 primer pair, 5′-GGGCTCCGCTTTATTTGCTC-3′ and 5′-CAGTTGAGATCCCCGAGACC-3′; RAD51 primer pair, 5′-GCTGGGGCGAAAACACAAG-3′ and 5′-GACTTCTCGCTCGAACCCAT-3′; and GAPDH primer pair, 5′- TACTAGCGGTTTTACGGGCG-3′ and 5′- AGGCTGCGGGCTCAATTTAT-3′. Non-quantitative PCRs and qPCRs were set up as described in 2.2. The immunoprecipitated DNAs were quantitated by standard curve method. The standard curve was generated with input chromatin DNA samples at concentrations of 50 ng, 5 ng, 0.5 ng and 0.05 ng per ul.

### Immunofluorescent staining

A2780/SRSF3si2 cells were grown on poly-L-lysine-coated glass coverslip in the presence or absence of Doxy for 3 days before subjected for staining. The cells were fixed in ice-cold methanol for 10 min followed by air-dry. Afterwards, the cells were blocked in 5 % normal donkey serum (Jackson ImmunoResearch, West Grove, PA) for 1 h before they were incubated with γ-H2AX antibody (Cell signaling Technology, Cat # 9718S, 1:400 dilution) for 1 h and then with Dylight 488-conjugated donkey anti-rabbit IgG (Jackson ImmunoResearch, Cat # 711-485-152, 1:200 dilution) for 45 min. The cells were rinsed in 1xPBS for three times after each incubation step. Finally, the coverslips were mounted on glass slides with VECTASHIELD Mounting Medium containing 4′, 6-Diamidino-2-phenylindole dihydrochloride (DAPI) (Vector Laboratories, Burlingame, CA).

### HR assay

The direct repeat green fluorescent protein (DR-GFP) reporter was used to measure HR activity in 293 T cells with or without SRSF3 knockdown. Briefly, 293 T cells grown in 12-well plate were infected at multiplicity of infection 5 with lentiviruses expressing SRSF3si1, SRSF3si2 or LUCsi for 12 h. Two days after infection, these cells were co-transfected with plasmids pDRGFP, pCBASceI (Addgene, Cambridge, MA) and pmCherry-N1 (Clontech Laboratories, Mountain View, CA) by calcium phosphate precipitation method [54]. The transfected cells were subjected to flow cytometric analysis for GFP-positive and mCherry-positive cells two days after transfection. The percentages of GFP-positive cells were normalized to the percentages of mCherry-positive cells before comparison.

### Statistical analysis

Unless otherwise stated, Student’s *t*-test was used in comparisons between samples. All tests were two-sided and p-values < 0.05 were considered significant.

### Accession numbers

The microarray data reported in this paper were deposited in Gene Expression Omnibus (GEO) database. The accession number is GSE71745.

## Additional files

Additional file 1: Table S1. Expression-altered genes in SRSF3-knockdown cells. (XLSX 120 kb) Additional file 2: Table S2. Splicing-altered genes in SRSF3-knopckdown cells. (XLSX 164 kb) Additional file 3: Figure S1-S7. Supplementary data. (PPTX 433 kb)

## Abbreviations

BRCA1: Breast cancer 1, early onset
BRIP1: BRCA1 interacting protein C-terminal helicase 1
ChIP: Chromatin immunoprecipitation
DAPI: 4′, 6-Diamidino-2-phenylindole dihydrochloride
Doxy: Doxycycline
DR-GFP: The direct repeat green fluorescent protein
DSB: DNA double-strand break
γ-H2AX: Phosphorylated histone H2AX
H3K4: Histone H3 lysine 4
H3K9: Histone H3 lysine 9
H3K27: Histone H3 lysine 27
HR: Homologous recombination
HRR: HR-mediated DNA damage repair
KMT2C: Lysine-specific methyltransferase 2C
LUCsi: Firefly luciferase siRNA
Neg IgG: Negative control IgG
NMD: Nonsense-mediated mRNA decay
PTC: Premature termination codons
qPCR: Quantitative RT-PCR
RAD51: RAD51 recombinase
RNApII: RNA polymerase II
RT-PCR: Reverse transcription PCR
SRSF3: Serine/arginine-rich splicing factor 3
SRSF3si1: SRSF3 siRNA1
SRSF3si2: SRSF3 siRNA2
TSS: Transcription start site
UPF: Up-frameshift
UPFsi: UPF1 siRNA
