# Supplementary material for: Serine/arginine-rich splicing factor 3 (SRSF3) regulates homologous recombination-mediated DNA repair
Source: Mol Cancer. 2015 Aug 19;14:158. doi: 10.1186/s12943-015-0422-1 (PMC4539922; doi:10.1186/s12943-015-0422-1)
Supplement: Additional file 3: Figure S1-S7. — Supplementary data. (PPTX 433 kb) [file 12943_2015_422_MOESM3_ESM.pptx]

## Slide 1
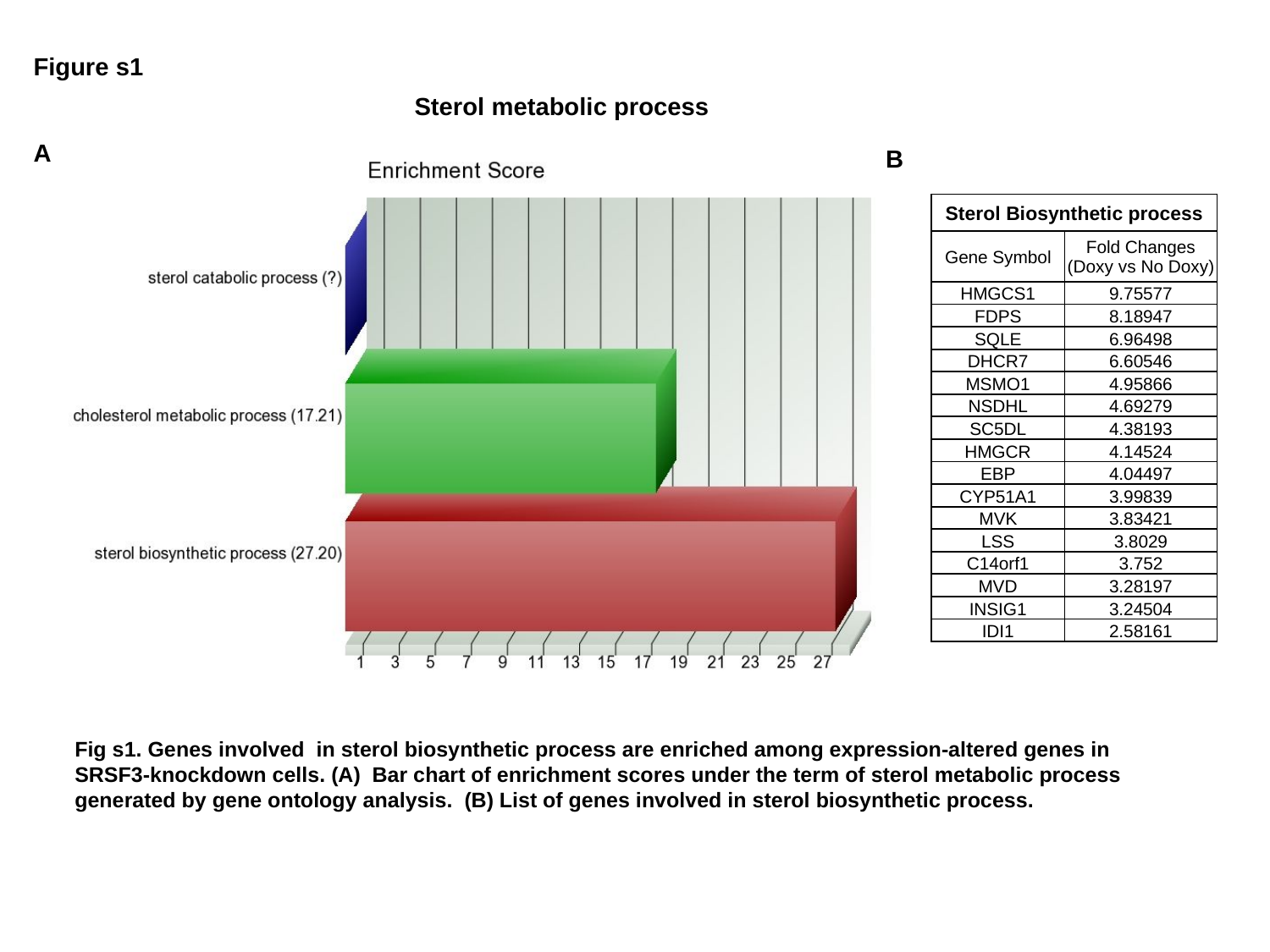

Figure s1
Sterol metabolic process
A
B
| Sterol Biosynthetic process | |
| --- | --- |
| Gene Symbol | Fold Changes (Doxy vs No Doxy) |
| HMGCS1 | 9.75577 |
| FDPS | 8.18947 |
| SQLE | 6.96498 |
| DHCR7 | 6.60546 |
| MSMO1 | 4.95866 |
| NSDHL | 4.69279 |
| SC5DL | 4.38193 |
| HMGCR | 4.14524 |
| EBP | 4.04497 |
| CYP51A1 | 3.99839 |
| MVK | 3.83421 |
| LSS | 3.8029 |
| C14orf1 | 3.752 |
| MVD | 3.28197 |
| INSIG1 | 3.24504 |
| IDI1 | 2.58161 |
Fig s1. Genes involved in sterol biosynthetic process are enriched among expression-altered genes in SRSF3-knockdown cells. (A) Bar chart of enrichment scores under the term of sterol metabolic process generated by gene ontology analysis. (B) List of genes involved in sterol biosynthetic process.

## Slide 2
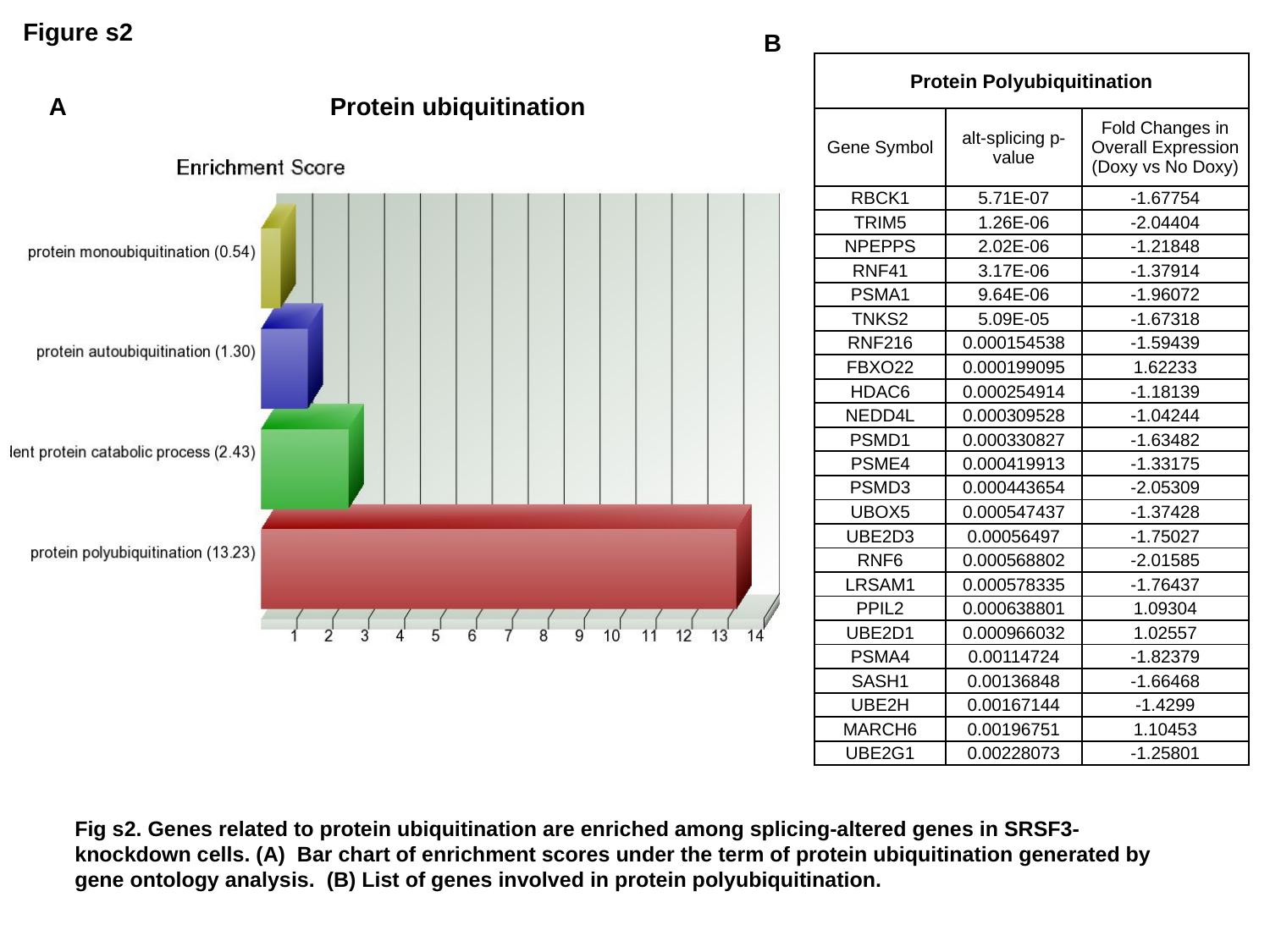

Figure s2
B
| Protein Polyubiquitination | | |
| --- | --- | --- |
| Gene Symbol | alt-splicing p-value | Fold Changes in Overall Expression (Doxy vs No Doxy) |
| RBCK1 | 5.71E-07 | -1.67754 |
| TRIM5 | 1.26E-06 | -2.04404 |
| NPEPPS | 2.02E-06 | -1.21848 |
| RNF41 | 3.17E-06 | -1.37914 |
| PSMA1 | 9.64E-06 | -1.96072 |
| TNKS2 | 5.09E-05 | -1.67318 |
| RNF216 | 0.000154538 | -1.59439 |
| FBXO22 | 0.000199095 | 1.62233 |
| HDAC6 | 0.000254914 | -1.18139 |
| NEDD4L | 0.000309528 | -1.04244 |
| PSMD1 | 0.000330827 | -1.63482 |
| PSME4 | 0.000419913 | -1.33175 |
| PSMD3 | 0.000443654 | -2.05309 |
| UBOX5 | 0.000547437 | -1.37428 |
| UBE2D3 | 0.00056497 | -1.75027 |
| RNF6 | 0.000568802 | -2.01585 |
| LRSAM1 | 0.000578335 | -1.76437 |
| PPIL2 | 0.000638801 | 1.09304 |
| UBE2D1 | 0.000966032 | 1.02557 |
| PSMA4 | 0.00114724 | -1.82379 |
| SASH1 | 0.00136848 | -1.66468 |
| UBE2H | 0.00167144 | -1.4299 |
| MARCH6 | 0.00196751 | 1.10453 |
| UBE2G1 | 0.00228073 | -1.25801 |
A
Protein ubiquitination
Fig s2. Genes related to protein ubiquitination are enriched among splicing-altered genes in SRSF3-knockdown cells. (A) Bar chart of enrichment scores under the term of protein ubiquitination generated by gene ontology analysis. (B) List of genes involved in protein polyubiquitination.

## Slide 3
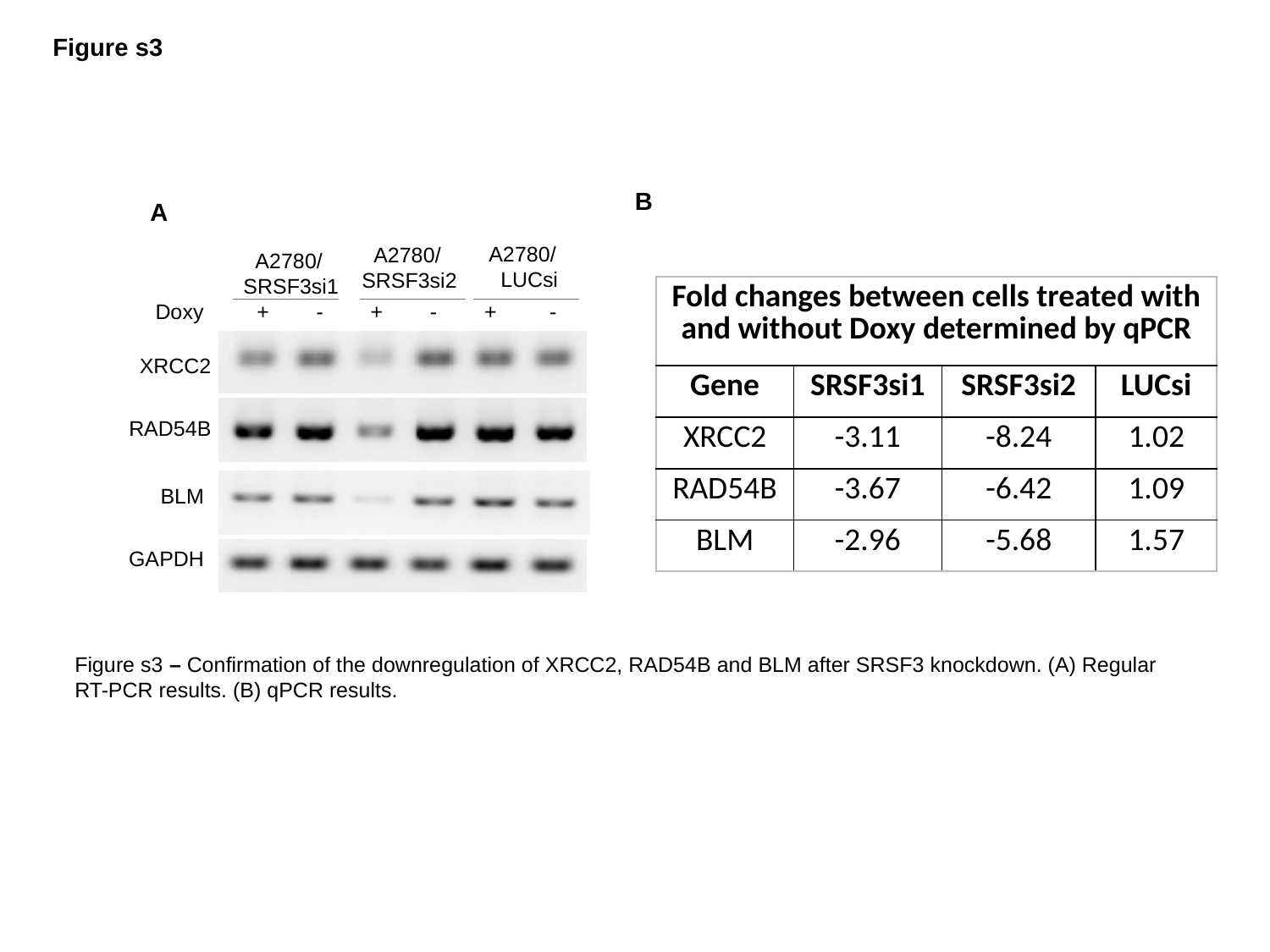

Figure s3
B
A
A2780/
 LUCsi
 A2780/
SRSF3si2
 A2780/
SRSF3si1
Doxy + - + - + -
XRCC2
RAD54B
BLM
GAPDH
| Fold changes between cells treated with and without Doxy determined by qPCR | | | |
| --- | --- | --- | --- |
| Gene | SRSF3si1 | SRSF3si2 | LUCsi |
| XRCC2 | -3.11 | -8.24 | 1.02 |
| RAD54B | -3.67 | -6.42 | 1.09 |
| BLM | -2.96 | -5.68 | 1.57 |
Figure s3 – Confirmation of the downregulation of XRCC2, RAD54B and BLM after SRSF3 knockdown. (A) Regular RT-PCR results. (B) qPCR results.

## Slide 4
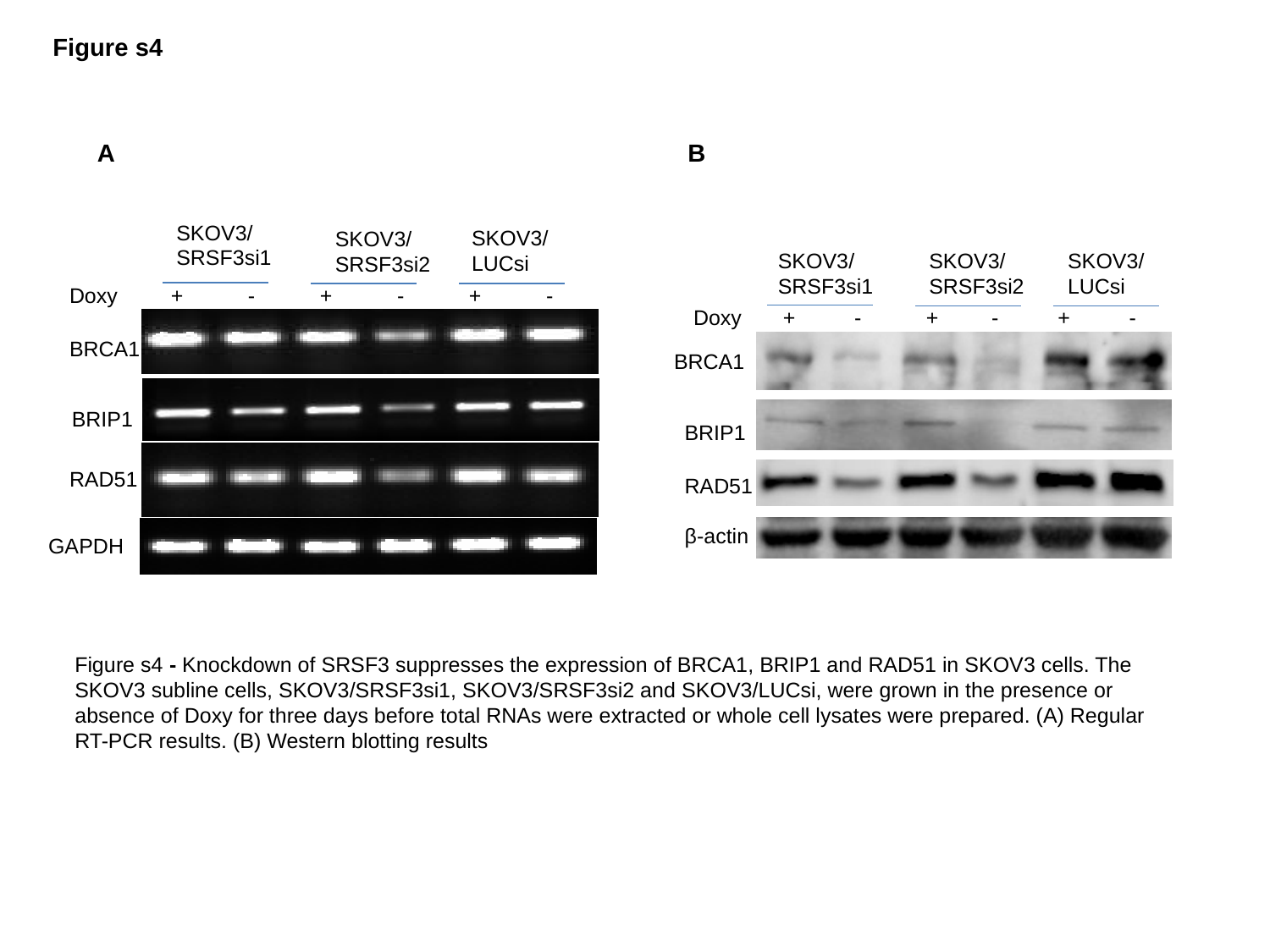

Figure s4
A
B
SKOV3/LUCsi
SKOV3/
SRSF3si1
SKOV3/
SRSF3si2
Doxy + - + - + -
BRCA1
BRIP1
RAD51
GAPDH
SKOV3/
SRSF3si2
SKOV3/ LUCsi
SKOV3/
SRSF3si1
Doxy + - + - + -
BRCA1
BRIP1
RAD51
β-actin
Figure s4 - Knockdown of SRSF3 suppresses the expression of BRCA1, BRIP1 and RAD51 in SKOV3 cells. The SKOV3 subline cells, SKOV3/SRSF3si1, SKOV3/SRSF3si2 and SKOV3/LUCsi, were grown in the presence or absence of Doxy for three days before total RNAs were extracted or whole cell lysates were prepared. (A) Regular RT-PCR results. (B) Western blotting results

## Slide 5
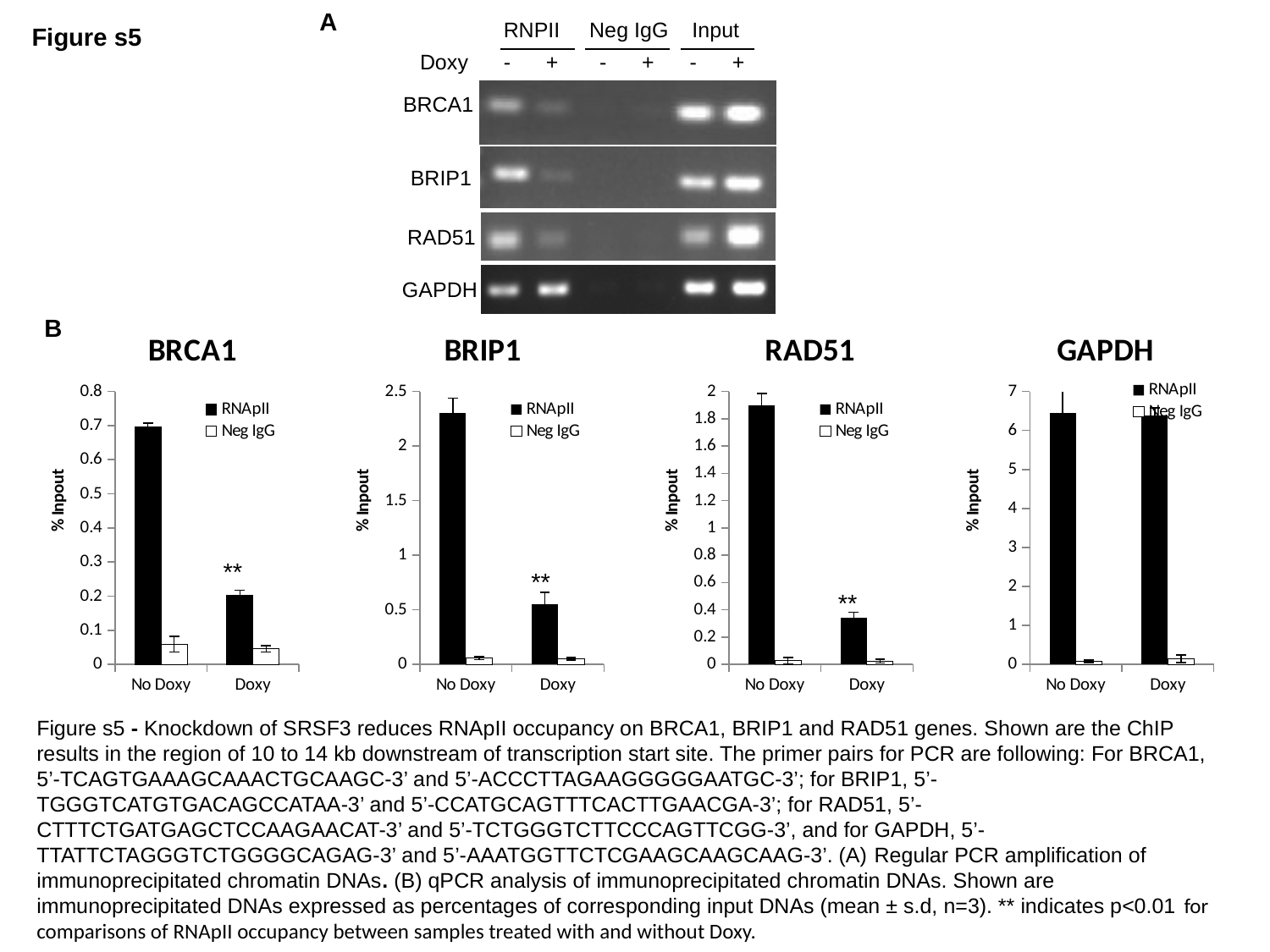

A
 RNPII Neg IgG Input
Doxy - + - + - +
BRCA1
BRIP1
RAD51
GAPDH
Figure s5
B
### Chart: BRCA1
| Category | | |
|---|---|---|
| No Doxy | 0.696154238421544 | 0.0593651133752296 |
| Doxy | 0.201264326798217 | 0.0454215633333 |
### Chart: BRIP1
| Category | | |
|---|---|---|
| No Doxy | 2.30363257256724 | 0.0573487032451267 |
| Doxy | 0.552456775394198 | 0.0523467124527341 |
### Chart: RAD51
| Category | | |
|---|---|---|
| No Doxy | 1.90134816412457 | 0.0257345257213457 |
| Doxy | 0.3392174842381 | 0.0241056447712348 |
### Chart: GAPDH
| Category | | |
|---|---|---|
| No Doxy | 6.45134529371547 | 0.078294156788823 |
| Doxy | 6.3925315676743 | 0.146173942802135 |**
**
**
Figure s5 - Knockdown of SRSF3 reduces RNApII occupancy on BRCA1, BRIP1 and RAD51 genes. Shown are the ChIP results in the region of 10 to 14 kb downstream of transcription start site. The primer pairs for PCR are following: For BRCA1, 5’-TCAGTGAAAGCAAACTGCAAGC-3’ and 5’-ACCCTTAGAAGGGGGAATGC-3’; for BRIP1, 5’-TGGGTCATGTGACAGCCATAA-3’ and 5’-CCATGCAGTTTCACTTGAACGA-3’; for RAD51, 5’-CTTTCTGATGAGCTCCAAGAACAT-3’ and 5’-TCTGGGTCTTCCCAGTTCGG-3’, and for GAPDH, 5’-TTATTCTAGGGTCTGGGGCAGAG-3’ and 5’-AAATGGTTCTCGAAGCAAGCAAG-3’. (A) Regular PCR amplification of immunoprecipitated chromatin DNAs. (B) qPCR analysis of immunoprecipitated chromatin DNAs. Shown are immunoprecipitated DNAs expressed as percentages of corresponding input DNAs (mean ± s.d, n=3). ** indicates p<0.01 for comparisons of RNApII occupancy between samples treated with and without Doxy.

## Slide 6
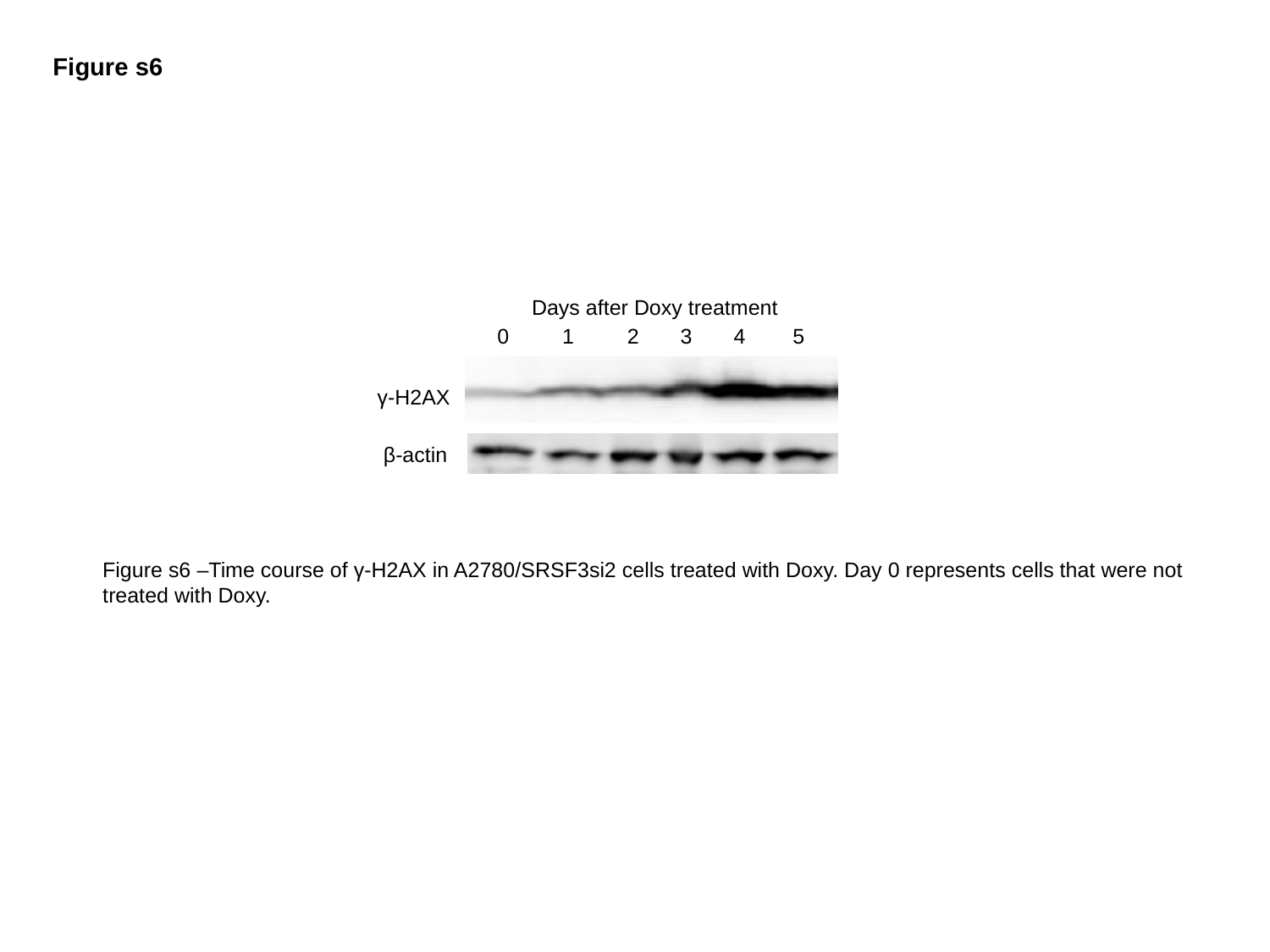

Figure s6
Days after Doxy treatment
0 1 2 3 4 5
γ-H2AX
β-actin
Figure s6 –Time course of γ-H2AX in A2780/SRSF3si2 cells treated with Doxy. Day 0 represents cells that were not treated with Doxy.

## Slide 7
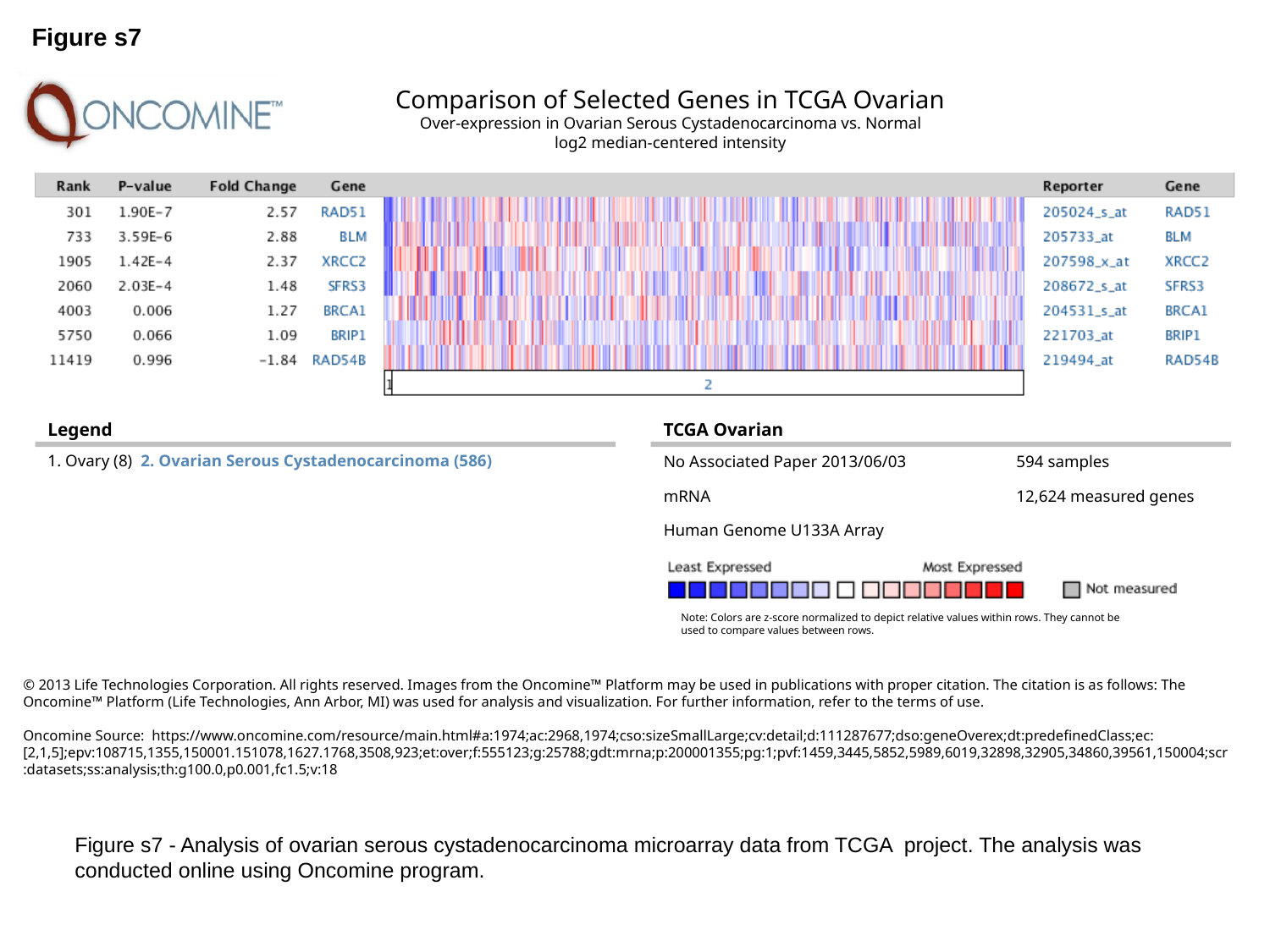

Figure s7
Comparison of Selected Genes in TCGA Ovarian
Over-expression in Ovarian Serous Cystadenocarcinoma vs. Normal
log2 median-centered intensity
Legend
TCGA Ovarian
1. Ovary (8) 2. Ovarian Serous Cystadenocarcinoma (586)
| No Associated Paper 2013/06/03 | 594 samples |
| --- | --- |
| mRNA | 12,624 measured genes |
| Human Genome U133A Array | |
Note: Colors are z-score normalized to depict relative values within rows. They cannot be used to compare values between rows.
© 2013 Life Technologies Corporation. All rights reserved. Images from the Oncomine™ Platform may be used in publications with proper citation. The citation is as follows: The Oncomine™ Platform (Life Technologies, Ann Arbor, MI) was used for analysis and visualization. For further information, refer to the terms of use.
Oncomine Source: https://www.oncomine.com/resource/main.html#a:1974;ac:2968,1974;cso:sizeSmallLarge;cv:detail;d:111287677;dso:geneOverex;dt:predefinedClass;ec:[2,1,5];epv:108715,1355,150001.151078,1627.1768,3508,923;et:over;f:555123;g:25788;gdt:mrna;p:200001355;pg:1;pvf:1459,3445,5852,5989,6019,32898,32905,34860,39561,150004;scr:datasets;ss:analysis;th:g100.0,p0.001,fc1.5;v:18
Figure s7 - Analysis of ovarian serous cystadenocarcinoma microarray data from TCGA project. The analysis was conducted online using Oncomine program.
